# Supplementary material for: Cognitive Dysfunction in Non-Alcoholic Fatty Liver Disease—Current Knowledge, Mechanisms and Perspectives
Source: J Clin Med. 2021 Feb 9;10(4):673. doi: 10.3390/jcm10040673 (PMC7916374; doi:10.3390/jcm10040673)
Supplement: Supplementary file 1 [file jcm-10-00673-s001.pdf]

`

Table S1. Description of neuropsychological tests applied in the studies outlined in Table 1 of the main manuscript.

	Neuropsychological Tests	Cognitive Domains Assessed	Test Execution
	Portosystemic Systemic Encephalopathy (PSE) test battery [2]		
**Felipo 2012 (Spain) [1]**	Digit Symbol Substitution Test (DST)	Visuospatial function and psychomotor speed	Number of correct substitutions of specific symbols each matched to a specific digit completed in 90 s
	Trailmaking A test (NCT-A)	Attention and psychomotor speed	Time (s) to connect randomly placed circles with numbers inside (from 1–25) in chronological order
	Trailmaking B test (NCT-B)	Executive function	Time (s) to connect randomly placed circles with numbers and letters inside (from 1–13; from A–L) in chronological and alphabetic order, alternating between numbers and letters
	Serial Dotting Test (SDT)	Attention and working memory	Time (s) to place a pencil dot inside 100 circles
	Line Tracing Test (LTT)	Visuospatial function	Time (s) to draw a line within a track, subtracted by errors where the pencil line touches the border of the track
**Seo 2016 (USA) [3]**	Simple Reaction Time Test (SRTT)	Psychomotor speed	Mean time (ms) to complete 40 simple visual-motor reaction time measurements
	Digit Symbol Substitution Test (SDST)	Visuospatial function and psychomotor speed	Number of correct substitutions of specific symbols each matched to a specific digit completed in 90 s
	Serial Digit Learning Test (SDLT)	Memory and attention	Sum of errors when asked to memorize a series of 8 digits in a maximum of 8 trials
**Takahashi 2017 (Japan) [4]**	Verbal Fluency Task (VFT)	Executive function, verbal fluency	Total number of (Japanese) words starting with a designated syllable listed in 20 s
**Tuttolomondo 2018 (Italy) [5]**	Mini Mental State Examination (MMSE) [6]	Visuospatial function, executive function, memory, attention, language, and orientation	10 simple neuropsychological subtests assessing global cognitive function (see [6] for details)
**Filipovic 2018 (Serbia) [7]**	Montreal Cognitive Assessment (MoCA) (www.mocatest.org)	Visuospatial function, executive function, memory, attention, language, and orientation	10 simple neuropsychological subtests assessing global cognitive function (see www.mocatest.org for details)
**Celikbilek 2018 (Turkey) [8]**	Montreal Cognitive Assessment (MoCA) (www.mocatest.org)	Visuospatial function, executive function, memory, attention, language, and orientation	10 simple neuropsychological subtests assessing global cognitive function (see www.mocatest.org for details)
**Weinstein 2018 (USA) [9]**	Consortium to Establish a Registry for Alzheimer Disease – Word Learning subset (CERAD-WL) [10]	Verbal memory (immediate and delayed recall)	Total number of words recalled over three trials, where the same 10 words are presented on paper in different orders (immediate recall), and total number of words recalled from the previous 10 words after a period of time (delayed recall)
	Animal Fluency Test (AFT) [11]	Executive function, verbal fluency	Total number of different animals listed in 60 s

`

	Digit Symbol Substitution Test (DSST)	Visuospatial function, psychomotor speed	Number of correct substitutions of specific symbols each matched to a specific digit completed in 90 s
**An 2019 (USA) [12]**	The Repeatable Battery for the Assessment of Neuropsychological Status (RBANS) [13]	Immediate and delayed memory, attention, language, and visuospatial memory	12 neuropsychological subtests yielding Index scores of the respective cognitive domains listed (see [13] for details)
**Weinstein 2019 (USA) [14]**	Wechsler Adult Intelligence Scale – Revised (WAIS-R) subtest: Logical memory delayed (LMd) [15]	Verbal memory (delayed recall)	Total number of words recalled after a period of time out of 10 words presented on paper
	Wechsler Adult Intelligence Scale – Revised (WAIS-R) subtests: Visual reproduction (VRd) [15]	Visual memory (delayed recall)	Number of successfully reproduced drawings of 3 geometric pictures of increasing complexity after a period of time
	Wechsler Adult Intelligence Scale – Revised (WAIS-R) subtest: The Similarities test (SIM) [15]	Abstract reasoning	Number of successful attempts in identifying the similarities/likeliness of two words (objects or concepts)
	Trailmaking A – B test (TrA-TrB)	Executive function	TrA: Time (s) to connect randomly placed circles with numbers inside (from 1–25) in chronological order<br>TrB: Time (s) to connect randomly placed circles with numbers and letters inside (from 1–13; from A–L) in chronological and alphabetic order, alternating between numbers and letters<br>Subtracting the two tests results in a more robust measure of executive function, adjusted for impaired psychomotor speed
	The Hooper Visual Organization Test (HVOT).	Visual perception	Time (min) to identify 30 objects represented in line drawings

The abbreviations for the neuropsychological tests applied in the separate articles are provided in parenthesis.

`

References

1. Felipo, V.; Urios, A.; Montesinos, E.; Molina, I.; Garcia-Torres, M.L.; Civera, M.; Olmo, J.A.; Ortega, J.; Martinez-Valls, J.; Serra, M.A., et al. Contribution of hyperammonemia and inflammatory factors to cognitive impairment in minimal hepatic encephalopathy. *Metabolic brain disease* **2012**, *27*, 51-58, doi:10.1007/s11011-011-9269-3.
2. Weissenborn, K.; Ennen, J.C.; Schomerus, H.; Rückert, N.; Hecker, H. Neuropsychological characterization of hepatic encephalopathy. *Journal of hepatology* **2001**, *34*, 768-773, doi:10.1016/s0168-8278(01)00026-5.
3. Seo, S.W.; Gottesman, R.F.; Clark, J.M.; Hernaez, R.; Chang, Y.; Kim, C.; Ha, K.H.; Guallar, E.; Lazo, M. Nonalcoholic fatty liver disease is associated with cognitive function in adults. *Neurology* **2016**, *86*, 1136-1142, doi:10.1212/wnl.0000000000002498.
4. Takahashi, A.; Kono, S.; Wada, A.; Oshima, S.; Abe, K.; Imaizumi, H.; Fujita, M.; Hayashi, M.; Okai, K.; Miura, I., et al. Reduced brain activity in female patients with non-alcoholic fatty liver disease as measured by near-infrared spectroscopy. *PloS one* **2017**, *12*, e0174169, doi:10.1371/journal.pone.0174169.
5. Tuttolomondo, A.; Petta, S.; Casuccio, A.; Maida, C.; Corte, V.D.; Daidone, M.; Di Raimondo, D.; Pecoraro, R.; Fonte, R.; Cirrincione, A., et al. Reactive hyperemia index (RHI) and cognitive performance indexes are associated with histologic markers of liver disease in subjects with non-alcoholic fatty liver disease (NAFLD): a case control study. *Cardiovasc Diabetol* **2018**, *17*, 28, doi:10.1186/s12933-018-0670-7.
6. Folstein, M.F.; Folstein, S.E.; McHugh, P.R. "Mini-mental state". A practical method for grading the cognitive state of patients for the clinician. *J Psychiatr Res* **1975**, *12*, 189-198, doi:10.1016/0022-3956(75)90026-6.
7. Filipovic, B.; Markovic, O.; Duric, V.; Filipovic, B. Cognitive Changes and Brain Volume Reduction in Patients with Nonalcoholic Fatty Liver Disease. *Can J Gastroenterol Hepatol* **2018**, *2018*, 9638797, doi:10.1155/2018/9638797.
8. Celikbilek, A.; Celikbilek, M.; Bozkurt, G. Cognitive assessment of patients with nonalcoholic fatty liver disease. *European journal of gastroenterology & hepatology* **2018**, *30*, 944-950, doi:10.1097/meg.0000000000001131.
9. Weinstein, A.A.; de Avila, L.; Paik, J.; Golabi, P.; Escheik, C.; Gerber, L.; Younossi, Z.M. Cognitive Performance in Individuals With Non-Alcoholic Fatty Liver Disease and/or Type 2 Diabetes Mellitus. *Psychosomatics* **2018**, *59*, 567-574, doi:10.1016/j.psym.2018.06.001.
10. Morris, J.C.; Heyman, A.; Mohs, R.C.; Hughes, J.P.; van Belle, G.; Fillenbaum, G.; Mellits, E.D.; Clark, C. The Consortium to Establish a Registry for Alzheimer's Disease (CERAD). Part I. Clinical and neuropsychological assessment of Alzheimer's disease. *Neurology* **1989**, *39*, 1159-1165, doi:10.1212/wnl.39.9.1159.
11. Campagna, F.; Montagnese, S.; Ridola, L.; Senzolo, M.; Schiff, S.; De Rui, M.; Pasquale, C.; Nardelli, S.; Pentassuglio, I.; Merkel, C., et al. The animal naming test: An easy tool for the assessment of hepatic encephalopathy. *Hepatology (Baltimore, Md.)* **2017**, *66*, 198-208, doi:10.1002/hep.29146.
12. An, K.; Starkweather, A.; Sturgill, J.; Salyer, J.; Sterling, R.K. Association of CTRP13 With Liver Enzymes and Cognitive Symptoms in Nonalcoholic Fatty Liver Disease. *Nurs Res* **2019**, *68*, 29-38, doi:10.1097/nnr.0000000000000319.
13. Randolph, C.; Tierney, M.C.; Mohr, E.; Chase, T.N. The Repeatable Battery for the Assessment of Neuropsychological Status (RBANS): preliminary clinical validity. *J Clin Exp Neuropsychol* **1998**, *20*, 310-319, doi:10.1076/jcen.20.3.310.823.
14. Weinstein, G.; Davis-Plourde, K.; Himali, J.J.; Zelber-Sagi, S.; Beiser, A.S.; Seshadri, S. Non-alcoholic fatty liver disease, liver fibrosis score and cognitive function in middle-aged adults: The Framingham Study. *Liver international : official journal of the International Association for the Study of the Liver* **2019**, *39*, 1713-1721, doi:10.1111/liv.14161.
15. Wechsler, D. *WAIS-R : manual : Wechsler adult intelligence scale--revised*; Harcourt Brace Jovanovich [for] Psychological Corp.: New York, NY, 1981.
